# Comparison of Body Surface Area and Optimized Multivariate Allometric Model Approaches in Correcting Echocardiographic Aortic Dimensions for Physiological Variances: An International and Multicenter Study (CITED I)

**DOI:** 10.1002/mco2.70788

**Published:** 2026-05-29

**Authors:** Guihua Yao, Xiaoxia Hu, Xiangyun Chen, Xueying Zeng, Francesco Ferrara, Andreina Carbone, Salvatore Rega, Monica Franzese, Pin Sun, Mei Zhang, Olga Vriz, Cheng Zhang, Eduardo Bossone, Yun Zhang

**Affiliations:** ^1^ State Key Laboratory For Innovation and Transformation of Luobing Theory; Key Laboratory of Cardiovascular Remodeling and Function Research, Department of Cardiology , Chinese Ministry of Education, Chinese National Health Commission Chinese Academy of Medical Sciences and Shandong Province Qilu Hospital of Shandong University Jinan China; ^2^ Department of Cardiology Qilu Hospital of Shandong University (Qingdao) Qingdao China; ^3^ School of Mathematical Sciences Ocean University of China Qingdao China; ^4^ Heart Department University Hospital of Salerno Salerno Italy; ^5^ Department of Public Health University of Naples “Federico II” Naples Italy; ^6^ Unit of Cardiology University of Campania “Luigi Vanvitelli” Naples Italy; ^7^ IRCCS SYNLAB SDN Naples Italy; ^8^ Department of Echocardiography The Affiliated Hospital of Qingdao University Qingdao China; ^9^ Department of Cardiology and Emergency San Antonio Hospital San Daniele Del Friuli Udine Italy; ^10^ Heart Centre of Excellence King Faisal Specialist Hospital & Research Center Riyadh Saudi Arabia

**Keywords:** aortic dimensions, body surface area, isometric and allometric scaling, optimized multivariate allometric model, physiological variance

## Abstract

Aortic dimensions differ between ethnic groups. To correct the ethnicity‐related differences in aortic dimensions, Ao‐a, Ao‐s, and Ao‐asc were measured by echocardiography in a total of 1820 Chinese and Italian healthy adults. The correction equations based on an optimized multivariate allometric model (OMAM) were constructed and tested, and the correction efficacies of the two approaches of body surface area (BSA) and OMAM were compared. The aortic dimensions were found to vary with physiological variables and ethnicities (all *p* < 0.05). Correction using BSA eliminated neither the differences in aortic dimensions across study populations nor the residual correlations with physiological variables (all *p* < 0.05). In contrast, indexation with OMAM equations eliminated both the ethnicity‐related differences and residual correlations (all |*r*| < 0.20 and *p* > 0.05). The success rate of the BSA approach was 0%, while that of the OMAM approach was 100% for correcting all aortic dimensions in Chinese, Italian, and combined populations, respectively. In conclusion, the OMAM approach is superior to BSA approach in correcting the variations in aortic dimensions. Using OMAM as a novel indexing tool may facilitate establishing universal cutoffs between normal and abnormal aortic dimensions among different ethnic populations in the world.

## Graphical abstract

Ao‐a, Ao‐s, and Ao‐asc were measured by echocardiography in 1820 gender‐ and age‐matched Chinese and Italian healthy adults. Correction equations based on an optimized multivariate allometric model (OMAM) and BSA approaches were constructed in group A and tested in group B at a ratio of 7:3. The correction efficacy was 0% for BSA and 100% for OMAM, respectively.

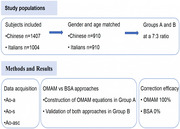

## 1 Introduction

Enlargement of the aortic root dimensions (ARD) is common in clinical practice. If severe, it is associated with aortic regurgitation, rupture, and dissection [1]. To detect and prevent aortic root dilation early, it is essential to acquire the normal values of the ARD in healthy adults to distinguish normality from abnormality [2]. Two‐dimensional echocardiography is commonly used to measure ARD. Many studies have demonstrated that ARD is not a fixed value in healthy adults but varies with several physiological variables, including gender, age, weight, and height [3, 4]. In an attempt to correct the effects of these physiological variables on the echocardiographic measurement of ARD, the American Society of Echocardiography (ASE) and the European Association of Cardiovascular Imaging (EACVI) have recommended an isometric model by dividing ARD measurements by body surface area (BSA) [5]. This approach assumes that a linear relationship exists between aortic diameters and BSA. However, the formula for estimating BSA, developed in 1916, was based on measurements of body weight and height in only nine patients and did not take into account the effects of gender and age [6].

Our previous study demonstrated that the use of BSA for indexation did not eliminate the effects of gender, age, and body size variables on cardiac measurements. Instead, it even increased the residual correlations between the BSA‐corrected values and body size variables in healthy adults [7]. In addition, the World Alliance of Societies of Echocardiography (WASE) study found significant ethnicity‐related differences in echocardiographic aortic dimensions, particularly between Asian and Caucasian populations, even after indexation with BSA [8]. This finding was highly consistent with our recent findings [9]. Unfortunately, due to the lack of a more accurate correction method, BSA is still recommended by international guidelines as a tool to correct the effects of physiological variables on echocardiographic measurements, despite its known limitations.

During the past two decades, many investigators have attempted to explore optimal approaches for correcting the effects of physiologic variables on echocardiographic aortic dimensions [10]. One important finding was that there was a nonlinear correlation between aortic dimensions and body size variables, the so‐called allometric correlation [11, 12]. Recently, our group developed an optimized multivariate allometric model (OMAM) to correct the effects of gender, age, and body size variables on echocardiographic measurements of 34 two‐dimensional echocardiographic parameters in 1224 healthy Chinese adults. We found that the success rate of correction was 100% using OMAM but only 11% using BSA indexation [7].

However, it remains unclear whether OMAM can eliminate the effects of physiological variables on ARD among different ethnic populations. Therefore, the purposes of this study were as follows: (1) to examine the differences in echocardiographic ARD between Chinese and Italian healthy adults, who represent typical Asian and Caucasian populations; (2) to assess whether the BSA and OMAM approaches could eliminate the differences in echocardiographic aortic root measurements between the Chinese and Italian populations; and (3) to establish the reference values of the OMAM‐corrected ARD in the Chinese and Italian populations. Our results showed that the aortic dimensions varied with gender, age, and ethnic groups, and these differences were not eliminated using the BSA approach but successfully removed with the OMAM approach. These results may facilitate establishment of a universal cutoff between normal and abnormal ARD among different ethnic populations in the world.

## 2 Results

### 2.1 Study Population

A total of 910 Chinese and 910 Italian subjects were finally selected for data analysis. An independent samples unpaired *t*‐test was performed to analyze the differences between genders and between Chinese and Italians. The demographic characteristics of the study population was listed in Table S1. There was no significant difference in age, gender ratio, and diastolic blood pressure (DBP) between the Chinese and Italian populations (all *p* > 0.05), while the mean values of height, weight, BSA, body mass index (BMI), and systolic blood pressure (SBP) were higher in the Italian than in the Chinese population (all *p* < 0.001). These results demonstrated a notable difference in body size between the Chinese and Italian populations.

### 2.2 Effects of Gender and Age on Aortic Dimensions in the Chinese Population

To examine the effects of gender and age on aortic dimensions in the Chinese population, all subjects in the Chinese population stratified by gender and age were divided into three age groups including young, middle‐aged, and elderly groups according to the WASE study [8]. An independent samples unpaired *t*‐test was conducted to compare the differences in aortic measurements between genders, and one‐way analysis of variance (ANOVA) was applied to compare the mean values among the three age groups. As shown in Table S2, the mean values of the aortic dimensions at three cross‐sectional levels were consistently higher in men than in women in the entire Chinese population and in the three age groups except for aortic annulus (Ao‐a) in the old age group (all *p *< 0.05). However, the mean values of Ao‐a, sinuses of Valsalva (Ao‐s), and proximal ascending aorta (Ao‐asc) were paradoxically higher in women than in men in the entire Chinese population and in the young and middle age groups (all *p *< 0.05) when the ARD were corrected by BSA, suggesting a marked overcorrection by BSA. In addition, all three parameters of the aortic dimensions increased significantly with age except for Ao‐a in men, and these age‐dependent differences became more prominent after indexation with BSA (all *p *< 0.05). These results demonstrated that gender and age exerted significant effects on the ARD and BSA is not ideal tool to correct for these effects in the Chinese population.

### 2.3 Effects of Gender and Age on Aortic Dimensions in the Italian Population

To clarify the effects of gender and age on aortic dimensions in the Italian population, an independent samples unpaired *t*‐test and ANOVA were respectively used to compare gender differences in aortic dimensions and differences among the three age groups in the Italian population stratified by gender and age. As presented in Table S3, the mean values of Ao‐a, Ao‐s, and Ao‐asc were substantially higher in men than in women in the entire Italian population and in the three age groups (all *p *< 0.05). However, these values became higher in women than in men in the entire Italian population (all *p *< 0.05) when the ARD were corrected by BSA, again suggesting an overcorrection by BSA (all *p *< 0.05). In addition, all three aortic dimensions increased with age and these differences remained significant after indexation with BSA (all *p *< 0.05). These results indicated that gender and age were important factors affecting the ARD and BSA was not a successful method to correct for these effects in the Italian population.

### 2.4 Comparison of the Aortic Dimensions Between the Chinese and Italian Populations

To compare the aortic dimensions between the Chinese and Italian populations, the Chinese and Italian populations were stratified by gender and age, and the results were shown in Table 1. The mean value of Ao‐a was significantly lower whereas those of Ao‐s and Ao‐asc were higher in the Italian population than in the Chinese population in both genders and most age groups (*p *< 0.05). As gender and age distributions were well balanced between the Chinese and Italian populations after propensity score analysis, the difference in the aortic dimensions between the two populations was mainly attributable to the ethnic discrepancy besides potential genetic and environmental confounders. However, except for Ao‐s/BSA in men and Ao‐asc/BSA in both genders, the ethnic differences remained significant even after normalization to BSA (*p *< 0.05). The results indicated that there was an ethnic discrepancy in aortic sizes between the Chinese and Italian populations and normalization to BSA did not remove the effect of race.

**TABLE 1:** Comparisons of aorta dimensions stratified by gender and age between the Chinese and Italian populations.

Aortic size	Gender	Chinese (*n* = 910)				Italian (*n* = 910)			
		8–40 y (*n* = 345)	41–65 y (*n* = 495)	65 y (*n* = 70)	Total (*n* = 910)	18–40 y (*n* = 345) (*p* value)	41–65 y (*n* = 495) (*p* value)	>65 y (*n* = 70) (*p* value)	Total (*n* = 910) (*p* value)
Ao‐a (mm)	Men	21.1 ± 2.2	21.5 ± 2.5	21.5 ± 2.1	21.4 ± 2.4	20.6 ± 2.0 (0.044)	21.0 ± 2.0 (0.016)	22.5 ± 2.0 (0.066)	21.0 ± 2.0 (0.012)
	Women	19.2 ± 2.1	20.0 ± 2.4	20.5 ± 2.6	19.7 ± 2.3	18.4 ± 1.5 (<0.001)	18.9 ± 1.5 (<0.001)	19.6 ± 1.6 (0.064)	18.8 ± 1.6 (<0.001)
Ao‐s (mm)	Men	28.8 ± 2.8	30.8 ± 3.2	31.4 ± 2.6	30.0 ± 3.2	30.4 ± 3.2 <0.001	32.8 ± 3.2 (<0.001)	35.0 ± 3.4 (<0.001)	32.0 ± 3.5 (<0.001)
	Women	26.5 ± 3.2	28.1 ± 3.1	28.0 ± 3.3	27.5 ± 3.2	26.7 ± 2.4 (0.471)	29.4 ± 2.7 (<0.001)	31.3 ± 3.3 (<0.001)	28.5 ± 3.0 (<0.001)
Ao‐asc (mm)	Men	26.4 ± 3.1	28.4 ± 3.7	30.0 ± 2.8	27.7 ± 3.6	27.3 ± 3.1 (0.007)	30.9 ± 3.5 (<0.001)	33.0 ± 3.8 (0.001)	29.7 ± 3.9 (<0.001)
	Women	24.4 ± 3.5	26.6 ± 3.3	26.5 ± 3.0	25.8 ± 3.5	25.2 ± 2.6 (<0.001)	28.3 ± 2.6 (<0.001)	30.4 ± 3.3 (<0.001)	27.3 ± 3.2 (<0.001)
Ao‐a/BSA (mm/m^2^)	Men	11.6 ± 1.4	12.1 ± 1.6	12.5 ± 1.4	11.9 ± 1.5	10.7 ± 1.5 (<0.001)	11.0 ± 1.2 (<0.001)	11.9 ± 1.3 (0.072)	11.0 ± 1.3 (<0.001)
	Women	12.2 ± 1.4	12.7 ± 1.6	13.4 ± 1.7	12.6 ± 1.6	11.2 ± 1.2 (<0.001)	11.3 ± 1.1 (<0.001)	11.9 ± 1.1 (<0.001)	11.3 ± 1.1 (<0.001)
Ao‐s/BSA (mm/m^2^)	Men	15.8 ± 1.6	17.3 ± 2.0	18.3 ± 1.5	16.8 ± 2.0	15.8 ± 2.2 (0.800)	17.2 ± 2.0 (0.747)	18.6 ± 2.5 (0.638)	16.8 ± 2.2 (0.957)
	Women	16.9 ± 2.2	17.8 ± 2.0	18.4 ± 2.7	17.5 ± 2.2	16.2 ± 1.8 (0.002)	17.6 ± 1.9 (0.376)	18.9 ± 2.0 (0.355)	17.2 ± 2.0 (0.035)
Ao‐asc/BSA (mm/m^2^)	Men	14.5 ± 1.7	15.9 ± 2.1	17.5 ± 1.6	15.5 ± 2.1	14.2 ± 2.1 (0.226)	16.2 ± 2.1 (0.195)	17.5 ± 2.3 (0.924)	15.5 ± 2.4 (0.802)
	Women	15.5 ± 2.2	16.9 ± 2.2	17.4 ± 2.2	16.4 ± 2.3	15.3 ± 1.9 (0.278)	17.0 ± 1.8 (0.412)	18.4 ± 2.0 (0.041)	16.5 ± 2.1 (0.558)

Abbreviations: Ao‐a, aortic annular diameter; Ao‐s, aortic sinus diameter; Ao‐asc, proximal ascending aortic diameter; BSA, body surface area.

*p* Value, in comparison with the Chinese population in the corresponding total, gender, and age groups

### 2.5 Demographic Characteristics in Group A and Group B in Chinese, Italian, and Combined Populations

To develop and validate OMAM equations in different populations, the Chinese, Italian, and combined Chinese–Italian populations were each randomly divided at a ratio of 7:3 into group A for the construction of OMAM equations and group B for the validation of OMAM equations. As listed in Table S4, there were no significant differences in age, height, weight, BMI, BSA, SBP, and DBP between group A and group B (all *p *> 0.05), which indicated that the grouping was completely randomized, and the demographic characteristics of group A and group B were comparable.

### 2.6 Construction and Validation of the OMAM Equations in Chinese Population

To construct and validate OMAM equations in the Chinese population, a stepwise multivariable linear regression analysis was performed to determine the variables entering the formula, as well as the values of scaling constants and exponents. The OMAM equations of Yp for three aortic dimensions were as follows: Ao‐a = 9.593 × 0.943^(man = 0, woman = 1)^ × age^0.054^ × weight^0.142^, Ao‐s = 8.085 × 0.949^(man = 0, woman = 1)^ × age^0.113^ × weight^0.210^, and Ao‐asc = 5.028 × 0.966^(man = 0, woman = 1)^ × age^0.161^ × weight^0.261^. As shown in Table 2, the values of Ao‐a, Ao‐s, and Ao‐asc were significantly correlated with age, height, weight, BSA, and BMI in group B of the Chinese population (*r *= 0.169–0.322, all *p *< 0.05). After indexation with BSA, these correlations became more significant (*r *= −0.451–0.315, all *p *< 0.05), with the negative correlations indicating an overcorrection. In contrast, after dividing the values of Ao‐a, Ao‐s, and Ao‐asc by their respective Yp, the derived Yc values were close to 1. These Yc values were highly correlated with the uncorrected values (*r *= 0.848–0.920, all *p *< 0.001) but were not significantly or biologically correlated with age, height, weight, BSA, and BMI in group B of the Chinese population (all |*r*| < 0.20 and *p *> 0.05), which fully met the predefined criteria for successful correction. These results suggested that the OMAM approach could completely remove the effects of biometric variables on aortic measurements in the Chinese population.

**TABLE 2:** Correlation of uncorrected and corrected aortic dimensions with physiological variables in group B of the Chinese population.

Aortic size	Mean values (mean ± SD)	Correlations with aortic size (*r, p* values)					
		Uncorrected value	Age	Height	Weight	BSA	BMI
Ao‐a (mm)	20.411 ± 2.538	−	0.169, 0.005	0.245, <0.001	0.311, <0.001	0.305, <0.001	0.240, <0.001
Ao‐s (mm)	28.629 ± 3.637	−	0.185, 0.002	0.259, <0.001	0.322, <0.001	0.318, <0.001	0.241, <0.001
Ao‐asc (mm)	26.625 ± 3.592	−	0.218, <0.001	0.181, 0.003	0.289, <0.001	0.265, <0.001	0.272, <0.001
Ao‐a/BSA (mm/m^2^)	12.193 ± 1.618	0.707, <0.001	0.274, <0.001	−0.442, <0.001	−0.419, <0.001	−0.451, <0.001	−0.201, 0.001
Ao‐s/BSA (mm/m^2^)	17.099 ± 2.310	0.713, <0.001	0.293, <0.001	−0.421, <0.001	−0.399, <0.001	−0.430, <0.001	−0.194, 0.001
Ao‐asc/BSA (mm/m^2^)	15.909 ± 2.293	0.750, <0.001	0.315, <0.001	−0.450, <0.001	−0.384, <0.001	−0.431, <0.001	−0.138, 0.023
Ao‐a/(9.593 × 0.943^(man = 0, woman = 1)^ × age^0.054^ × weight^0.142^)	0.999 ± 0.116	0.920, <0.001	0.072, 0.238	−0.034, 0.579	−0.014, 0.824	−0.021, 0.726	0.018, 0.770
Ao‐s/(8.085 × 0.949^(man = 0, woman = 1)^ × age^0.113^ × weight^0.210^)	1.002 ± 0.116	0.868, <0.001	−0.065, 0.283	−0.021, 0.725	−0.061, 0.318	−0.050, 0.414	−0.072, 0.237
Ao‐asc/(5.028 × 0.966^(man = 0, woman = 1)^ × age^0.161^ × weight^0.261^)	1.004 ± 0.127	0.848, <0.001	−0.133, 0.028	−0.051, 0.404	−0.081, 0.184	−0.074, 0.222	−0.071, 0.244

Abbreviations: Ao‐a, aortic annular diameter; Ao‐s, aortic sinus diameter; Ao‐asc, proximal ascending aortic diameter; BSA, body surface area; BMI, body mass index.

### 2.7 Construction and Validation of the OMAM Equations in the Italian Population

To construct and validate OMAM equations in the Italian population, the OMAM equations of Yp were developed for three aortic diameters in the Italian population by performing a stepwise multivariable linear regression analysis with the following results: Ao‐a = 1.042 × 0.944^(man = 0, woman = 1)^ × age^0.054^ × height^0.472^ × weight^0.081^, Ao‐s = 2.268 × 0.925^(man = 0, woman = 1)^ × age^0.164^ × height^0.317^ × weight^0.090^, and Ao‐asc  = 6.385 × 0.956^(man = 0, woman = 1)^ × age^0.204^ × weight^0.175^. As shown in Table 3, the values of Ao‐a, Ao‐s, and Ao‐asc correlated significantly with age, height, weight, BSA, and BMI in group B of the Italian population (*r *= 0.126–0.503, *p *< 0.05), except for the relation between Ao‐a and age (*r *= 0.110, *p *= 0.070). After indexation with BSA, these correlations remained significant (*r *= −0.614–0.511, *p *< 0.001), with the negative correlations again indicating an overcorrection, except for the relations between Ao‐s/BSA and BMI and between Ao‐asc/BSA and BMI. On the other hand, after dividing the values of Ao‐a, Ao‐s, and Ao‐asc by their respective Yp, the derived Yc values correlated highly with the uncorrected values (*r *= 0.768–0.807, all *p *< 0.001) but did not correlate significantly with age, height, weight, BSA, and BMI in group B of the Italian population (all |*r*| < 0.20 and *p *> 0.05), which fully met the predefined criteria for successful correction. These results demonstrated that the OMAM approach could remove the effects of biometric variables on aortic diameters in the Italian population.

**TABLE 3:** Correlation of uncorrected and corrected aortic dimensions with physiological variables in group B of the Italian population.

Aortic size	Mean values (mean ± SD)	Correlations with aortic size (*r, p* values)					
		Uncorrected value	Age	Height	Weight	BSA	BMI
Ao‐a (mm)	19.786 ± 2.012	−	0.110, 0.070	0.413, <0.001	0.457, <0.001	0.397, <0.001	0.261, <0.001
Ao‐s (mm)	29.884 ± 3.579	−	0.411, <0.001	0.278, <0.001	0.437, <0.001	0.329, <0.001	0.358, <0.001
Ao‐asc (mm)	28.266 ± 3.691	−	0.503, <0.001	0.126, 0.042	0.347, <0.001	0.221, <0.001	0.381, <0.001
Ao‐a/BSA (mm/m^2^)	11.125 ± 1.349	0.452, <0.001	0.195, 0.001	−0.348, <0.001	−0.421, <0.001	−0.614, <0.001	−0.266, <0.001
Ao‐s/BSA (mm/m^2^)	16.805 ± 2.301	0.582, <0.001	0.451, <0.001	−0.375, <0.001	−0.333, <0.001	−0.552, <0.001	−0.118, 0.052
Ao‐asc/BSA (mm/m^2^)	15.906 ± 2.480	0.665, <0.001	0.511, <0.001	−0.439, <0.001	−0.338, <0.001	−0.558, <0.001	−0.061, 0.329
Ao‐a/(1.042 × 0.944^(man = 0, woman = 1)^ × age^0.054^ × height^0.472^ × weight^0.081^)	1.008 ± 0.085	0.803, <0.001	0.066, 0.275	−0.099, 0.102	−0.041, 0.501	−0.069, 0.257	0.042, 0.495
Ao‐s/(2.268 × 0.925^(man = 0, woman = 1)^ × age^0.164^ × height^0.317^ × weight^0.090^)	0.996 ± 0.093	0.768, <0.001	0.077, 0.202	−0.068, 0.264	0.022, 0.715	−0.037, 0.541	0.098, 0.107
Ao‐asc/(6.385 × 0.956^(man = 0, woman = 1)^ × ·age^0.204^ × weight^0.175^)	1.001 ± 0.102	0.807, <0.001	0.055, 0.372	−0.017, 0.788	0.051, 0.413	−0.009, 0.880	0.088, 0.156

Abbreviations: Ao‐a, aortic annular diameter; Ao‐s, aortic sinus diameter; Ao‐asc, proximal ascending aortic diameter; BSA, body surface area; BMI, body mass index.

### 2.8 Construction and Validation of the OMAM Equations in the Combined Population

To develop and validate OMAM equations in the combined Chinese–Italian population, the OMAM equations of Yp were constructed for three aortic dimensions through a stepwise multivariable linear regression analysis as follows: Ao‐a = 2.948 × 0.928^(man = 0, woman = 1)^ × age^0.060^ × height^0.338^, Ao‐s = 2.689 × 0.950^(man = 0, woman = 1)^ × age^0.137^ × height^0.210^ × weight^0.198^, and Ao‐asc = 4.375 × 0.974^(man = 0, woman = 1)^ × age^0.180^ × weight^0.280^. As depicted in Table S5, the values of Ao‐a, Ao‐s, and Ao‐asc were significantly correlated with age, height, weight, BSA, and BMI in group B of the combined population (*r *= 0.144–0.412, all *p *< 0.001). After indexation with BSA, these correlations became more prominent (*r *= −0.567–0.410, all *p *< 0.05), with the negative correlations again indicating an overcorrection. By contrast, after dividing the values of Ao‐a, Ao‐s, and Ao‐asc by their respective Yp, the derived Yc values were highly correlated with the uncorrected values (*r *= 0.808–0.890, all *p *< 0.001) but were not significantly correlated with age, height, weight, BSA, and BMI in group B of the combined population (all |*r*| < 0.20 and *p *> 0.05), which fully met the predefined criteria for successful correction. These results again demonstrated the superior efficacy of OMAM over BSA.

### 2.9 Efficacy of OMAM Approach in Correcting Aortic Dimensions in Chinese, Italian, and Combined Populations

To compare the correction efficacy of OMAM and BSA approaches, the success rate of correction was calculated using the two methods. The OMAM‐corrected aortic dimensions stratified by gender were shown in Table 4. The OMAM‐corrected values (mean 0.987–1.016) of Ao‐a, Ao‐s, and Ao‐asc were similar between men and women in group B of the Chinese, Italian, and combined populations, respectively (all *p *> 0.05). These results demonstrated that the effect of gender on the aortic dimensions was successfully removed by indexation with the OMAM approach. Using the predefined criteria, the success rate of the BSA approach was 0%, while that of the OMAM approach was 100% for correcting Ao‐a, Ao‐s, and Ao‐asc in the Chinese, Italian, and combined populations, respectively, although such a high success rate might not be reproduced in populations other than Chinese and Italians.

**TABLE 4:** Efficacy of the OMAM approach for correcting the effect of gender on aortic dimensions in group B of the Chinese, Italian, and combined populations.

OMAM correction		Men	Women	*p* Value
Chinese population	Ao‐a/(9.593 × 0.943^(man = 0, woman = 1)^ × age^0.054^ × weight^0.142^)	0.987 ± 0.112	1.008 ± 0.118	0.131
	Ao‐s/(8.085 × 0.949^(man = 0, woman = 1)^ × age^0.113^ × weight^0.210^)	1.000 ± 0.104	1.003 ± 0.125	0.840
	Ao‐asc/(5.028 × 0.966^(man = 0, woman = 1)^ × age^0.161^ × weight^0.261^)	0.989 ± 0.122	1.016 ± 0.130	0.086
Italian population	Ao‐a/(1.042 × 0.944^(man = 0, woman = 1)^ × age^0.054^ × height^0.472^ × weight^0.081^)	1.006 ± 0.089	1.010 ± 0.082	0.704
	Ao‐s/(2.268 × 0.925^(man = 0, woman = 1)^ × age^0.164^ × height^0.317^ × weight^0.090^)	0.988 ± 0.097	1.002 ± 0.089	0.196
	Ao‐asc/(6.385 × 0.956^(man = 0, woman = 1)^ × age^0.204^ × weight^0.175^)	1.002 ± 0.112	1.000 ± 0.093	0.906
Combined population	Ao‐a/(2.948 × 0.928^(man = 0, woman = 1)^ × age^0.060^ × height^0.338^)	0.996 ± 0.101	1.010 ± 0.108	0.117
	Ao‐s/(2.689 × 0.950^(man = 0, woman = 1)^ × age^0.137^ × height^0.210^ × weight^0.198^)	0.992 ± 0.100	1.001 ± 0.109	0.294
	Ao‐asc/(4.375 × 0.974^(man = 0, woman = 1)^ × age^0.180^ × weight^0.280^)	0.995 ± 0.120	1.007 ± 0.114	0.217

Abbreviations: Ao‐a, aortic annular diameter; Ao‐s, aortic sinus diameter; Ao‐asc, proximal ascending aortic diameter; OMAM, optimized multivariate allometric model.

### 2.10 OMAM‐Corrected Reference Values of ARD

To establish the OMAM‐corrected reference values of ARD for the Chinese and Italian populations, the mean ± SD and 95% confidence intervals were listed in **Table** **5**. We developed a web‐based calculator to facilitate the clinical application of the OMAM approach. Users may input the values of age, gender, height, weight, and measured aortic dimensions for a given subject and the predicted and OMAM‐corrected values of Ao‐a, Ao‐s, and Ao‐asc will be provided automatically. The calculator is publicly available at the following link: https://xueyingzeng.github.io/OMAMaorta/. For a given subject, if the OMAM‐corrected value falls within the 95% confidence interval, it means his or her aortic diameter is normal. However, if the OMAM‐corrected value is below the lower limit or above the upper limit, it means his or her aortic diameter is decreased or increased.

**TABLE 5:** The reference values of OMAM‐corrected aortic dimensions for the Chinese and Italian populations (mean ± SD and 95% CI).

Aortic diameters	OMAM corrected values in Chinese population (*n* = 910)	OMAM‐corrected values in Italian population (*n* = 910)
Ao‐a	1.002 ± 0.112 0.794–1.246	1.007 ± 0.086 0.854–1.188
Ao‐s	1.003 ± 0.105 0.792–1.213	1.004 ± 0.090 0.834–1.185
Ao‐asc	1.006 ± 0.122 0.747–1.262	1.006 ± 0.099 0.802–1.195

Abbreviations: Ao‐a, aortic annular diameter; Ao‐s, aortic sinus diameter; Ao‐asc, proximal ascending aortic diameter; BSA, body surface area; BMI, body mass index; OMAM, optimized multivariate allometric model; SD, standard deviation; CI, confidence interval.

### 2.11 Reproducibility of Echocardiographic Measurements

**FIGURE 1:**
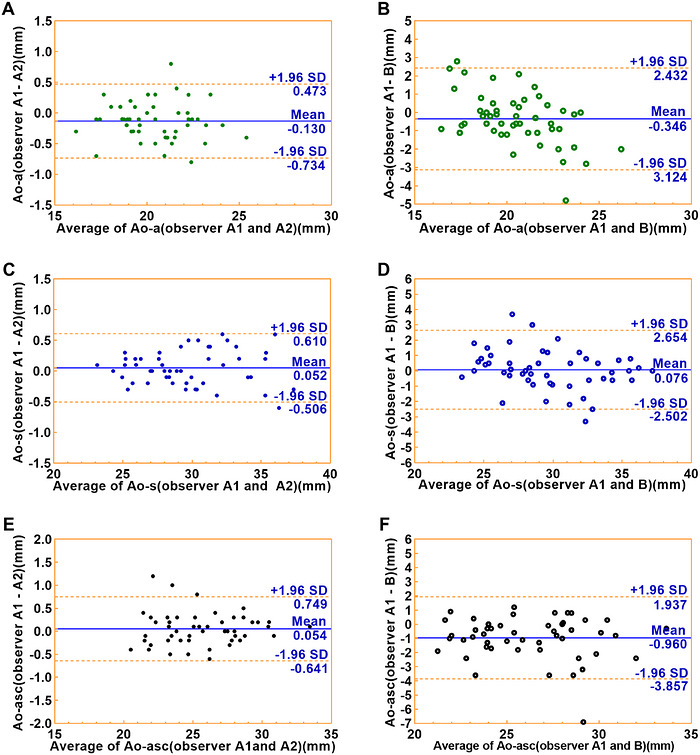
Bland–Altman plots of intra‐ and interobserver variability of aortic measurements. Panels (A) and (B): intra‐ and interobserver variability for Ao‐a; panels (C) and (D): intra‐ and interobserver variability for Ao‐s; panels (E) and (F): intra‐ and interobserver variability for Ao‐asc. Abbreviations: Ao‐a, aortic annular diameter; Ao‐s, aortic sinus diameter; Ao‐asc, proximal ascending aortic diameter.

To assess the consistency and stability of the aortic size measurements, the intraobserver and interobserver reproducibility of Ao‐a, Ao‐s, and Ao‐asc measurements was analyzed and presented in Figure 1. The intraclass correlation coefficients (ICCs) of intraobserver reproducibility for Ao‐a, Ao‐s, and Ao‐asc were 0.988, 0.997, and 0.993, respectively. For interobserver reproducibility, they were 0.813, 0.937, and 0.884, respectively (all *p *<  0.001). This indicated that the repeatability and agreement for measurements of aortic size were good to excellent.

## 3 Discussion

There were several important findings in the present study: (1) In both Chinese and Italian healthy populations, the ARD were consistently higher in men than in women and increased with age. Indexation with BSA did not eliminate these differences and even resulted in an overcorrection. (2) The ARD showed significant differences between the Chinese and Italian populations beyond the effects of gender and age. BSA indexation did not completely remove these ethnic differences. (3) In both Chinese and Italian populations, the ARD correlated significantly with age, height, weight, BSA, and BMI. After indexation with BSA, these correlations remained significant or even became negative. (4) The ARD corrected with specific OMAM equations developed from the Chinese, Italian, and combined Chinese–Italian populations correlated highly with the original measured values but did not correlate significantly or biologically with age, height, weight, BSA, and BMI in the three populations. (5) The between‐gender differences in the ARD were successfully removed by indexation with the OMAM equations in the Chinese, Italian, and combined populations. (6) Using predefined criteria, the success rate of correction was 0% for the BSA approach, while it was 100% for the OMAM approach in the Chinese, Italian, and combined populations. To the best of our knowledge, this was the first study to demonstrate the superiority of the OMAM over the BSA approach in eliminating the effects of physiological and ethnic variance on the echocardiographicA.

The effects of physiological variables on ARD have been recognized for many years [13]. These effects have to be eliminated to ensure a comparable measurement and unified cutoffs among different populations [11, 14]. The current echocardiographic guidelines recommend indexation of ARD with BSA, the so‐called isometric or linear scaling approach [5]. However, accumulating studies have consistently demonstrated that the BSA approach does not remove the impacts of physiological variables on ARD [11, 14, 15]. As demonstrated in the WASE study, all ARD were consistently larger in men than in women. Meanwhile, the BSA‐indexed aortic dimensions were significantly larger in women than in men, indicating an overcorrection by the BSA approach [8]. The WASE study also found that ARD were larger in older age groups in both genders. This trend persisted regardless of BSA or height indexation with the exception of the aortic annulus diameter, which appeared to be similar among all ages. More importantly, ethnicity‐related differences in ARD, especially between the Caucasian and Asian populations, were observed even after adjusting for BSA and in gender‐specific subgroups [8]. In the present study, we found that the ARD differed between genders and different ethnic populations, and correlated with age, height, weight, BSA, and BMI. These differences and correlations remained significant even after indexation with BSA, thus casting further doubt on the BSA approach. Based on these results, we believe that the BSA approach recommended by current guidelines should be abandoned, and a scientifically more solid indexation approach should be explored.

There is growing evidence supporting the application of allometric scaling of cardiac measurements to facilitate more accurate clinical decision‐making [11, 14]. A study by Oxborough et al. demonstrated that there was an allometric relationship between ARD and BSA, and allometric scaling of the order of BSA^0.6^ provided a body size‐independent index that was not influenced by age or gender [11]. In a large cohort of competitive athletes, Abulí et al. obtained body size‐independent ARD values using allometric scaling by BSA^0.578^ or by height^1.025^ and found that the residual correlations of BSA^0.578^‐corrected or height^1.025^‐corrected values with BSA (*r *= 0.063, *p *= 0.04) and height (*r *= 0.070, *p *= 0.001) were biologically insignificant [16]. The results indicated that although the body size effect on ARD was successfully removed using the allometric model by BSA^0.578^ or height^1.025^, the gender difference still remained. Another study by Oates et al. in a group of athletes indicated that allometric scaling using BSA or height produced body size‐independent ARD. However, the residual correlations of aortic dimensions with age and between‐gender differences were not mentioned in their study [15]. Recently, researchers from Spain explored age‐independent ARD in adolescent athletes. They found that the significant correlations between ARD and age were lost when indexed allometrically with BSA^0.5^, but whether gender‐independent and body size‐independent aortic values could be produced by applying the allometric scaling of BSA^0.5^ was not analyzed [17]. In summary, several previous studies applied allometric models with BSA or height in scaling ARD and produced body size‐independent values, demonstrating a superior correction efficacy of allometric models over the commonly used BSA approach. Unfortunately, the effects of other biometric variables such as gender, age, and ethnicity were not taken into account in their models, nor were residual correlations with these biometric variables or gender differences analyzed.

Previous studies showed that gender, age, and body size were the principal physiological determinants of ARD, which correlated allometrically with body size [11, 18]. Thus, it is inappropriate to scale aortic measurements using a single variate model, and a multivariate allometric model is theoretically more appropriate. A recent study by Mirea et al. demonstrated that including age, gender, height, and weight together in both the multivariate ratiometric model and the multivariate allometric model led to a significant improvement in the overall explained variance compared with a ratiometric model that included only BSA or both gender and age [19]. Both the multivariate ratiometric and allometric scaling methods produced negligible residual correlations of aortic measurements with anthropometric variables including BSA, weight, and height. However, their residual correlations with age and the difference between genders were not analyzed [19]. In the present study, the physiological variables that entered the OMAM equations in the Chinese population included gender, age, and weight. This is probably because the role of gender in the OMAM equations replaced that of height. By comparison, in the Italian population, the variables that entered the OMAM equations for correcting Ao‐a and Ao‐s included gender, age, height, and weight. This is likely due to the fact that height played an independent role in the OMAM equations apart from that of gender. Thus, in the combined Chinese–Italian population, the physiological variables that entered the OMAM equations were something in between. The equation for correcting Ao‐a included variables of gender, age, and height, and the equation for correcting Ao‐s included variables of gender, age, height, and weight. These results indicated that the variables entering the OMAM equation depended on the characteristics of the study population. Gender and age were the constant variables, while height and weight were changeable variables. Nonetheless, the application of the OMAM approach successfully removed the physiologic effects of gender, age, and body size variables on aortic dimensions in the Chinese, Italian, and combined populations, respectively. Most importantly, the success rate of the OMAM approach in correcting the effects of physiological variance on the ARD reached 100% in the Chinese, Italian, and combined populations. Thus, the OMAM approach could be applied in clinical practice not only in Asian but also in Caucasian populations.

This study has several limitations. First, the construction and computation of the OMAM equations are complex, and no specialized software is available in standard echo machines. To facilitate clinical application, we developed a web‐based calculator for OMAM‐correction. With this calculator, users can conveniently input the values of gender, age, height, weight, and measured aortic dimensions, and the predicted and OMAM‐corrected values of Ao‐a, Ao‐s, and Ao‐asc will be calculated automatically. The calculator is now publicly available at the following link to ensure easy access for clinical researchers and practitioners: https://xueyingzeng.github.io/OMAMaorta/. Second, since the physiological variables used in the OMAM equation depend on the characteristics of the enrolled subjects, the equations developed in this study may change in other populations. Third, only the Chinese and Italian populations were included in the present study, and the OMAM equations derived from these two populations might not be applicable to other ethnic groups. Nevertheless, the OMAM approach can be extrapolated to other Asian and Caucasian populations with reasonable accuracy. Finally, for the purpose of this study, only healthy and nonobese individuals were recruited. Since obese subjects are more vulnerable to vascular remodeling, future studies on constructing OMAM equations for indexing echocardiographic ARD in obese populations are warranted.

## 4 Conclusion

The ARD vary according to gender, age, weight, height, and ethnic groups in Chinese and Italian healthy populations. Indexation using BSA cannot eliminate these variations and even lead to over correction. In contrast, indexation with the OMAM approach successfully removes all these variations, demonstrating the superior efficacy of the OMAM approach over the BSA approach in indexing ARD in healthy subjects. Using OMAM as a novel indexing tool may facilitate establishing universal cutoffs between normal and abnormal ARD among different ethnic populations in the world.

## 5 Materials and Methods

### 5.1 Study Design and Populations

As the WASE study found that most racial differences in echocardiographic measurements lay between Asians and Caucasians [20], and because Chinese are representative of east Asians while Italian was one of the Caucasian populations showing the largest cardiovascular dimensions in the WASE study [20], two healthy adult populations were chosen from China and Italy for comparison in the present study. The Chinese and Italian study on Echocardiographic Dimensions (CITED) is an international, multicenter, and prospective study organized by Qilu Hospital of Shandong University, China, Qilu Hospital of Shandong University (Qingdao), China, and the Public Health Department, University of Naples “Federico II”, Italy. This study was approved by the independent local ethics committees (the approval number was 12144 for the Chinese study and n.84 r.p.s.o ‐ 2‐12‐2015 for the Italian study). The objective of the CITED study was to evaluate the OMAM approach for eliminating the differences in echocardiographic measurements of cardiovascular dimensions between the Chinese and Italian healthy populations and to establish the reference values of the OMAM‐corrected cardiovascular dimensions in the Chinese, Italian, and Chinese–Italian combined populations, respectively. The present report was the first part of the CITED study (CITED I). Two large populations were prospectively recruited. The first population was from the Echocardiographic Measurements in Normal Chinese Adults (EMINCA) study, which was conducted in 43 collaborating laboratories throughout the mainland of China from January 2012 to December 2012 [21]. The second population was from the Normal Values of Aortic Root Dimensions in Healthy Adults study, which as conducted in two hospitals in Italy from June 2007 to February 2014 [12, 22]. The inclusion criteria were as follows: (1) aged between 18 and 79 years; (2) having Chinese Han or Italian nationality; (3) having normal blood pressure (SBP < 140 mmHg, DBP < 90 mmHg); (4) having normal results on physical examination, electrocardiography, and two‐dimensional and Doppler echocardiography; (5) having no history of cardiovascular diseases. The exclusion criteria were: coronary artery disease, structural heart disease, heart failure, hypertension, stroke, hyperlipidemia, diabetes, and any other endocrine diseases, acute or chronic respiratory diseases, anemia, connective tissue disease, abnormal liver or renal function, obesity (BMI ≥ 30 kg/m^2^), abnormal electrocardiography results, valvular stenosis, more than mild valvular regurgitation, or wall motion abnormalities on echocardiographic recordings. Professional athletes, pregnant or lactating women, subjects addicted to alcohol or illicit drugs, and subjects with inadequate echocardiographic images were also excluded. A total of 1407 consecutive healthy Chinese volunteers and 1004 healthy Italian subjects who met the inclusion criteria and did not meet exclusion criteria constituted the research population of the present study. BSA was calculated using the formula of Du Bois and Du Bois [6].

### 5.2 Echocardiographic Image Acquisition

**FIGURE 2:**
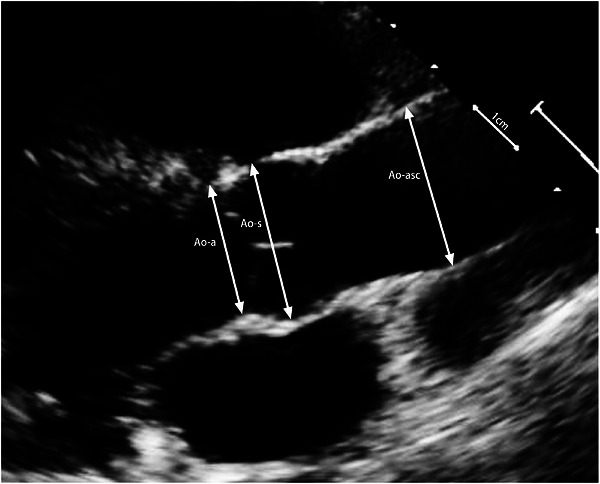
From the parasternal long‐axis view, aortic root diameters (annulus, sinuses of Valsalva, and proximal ascending aorta) were measured at end‐diastole using a leading‐edge to leading‐edge method.

Standard transthoracic two‐dimensional echocardiography was performed on all subjects according to the ASE/EACVI guidelines [5, 23]. The offline image analysis was carried out by two independent echocardiographic experts: Guihua Yao from Qilu Hospital of Shandong University for the Chinese population study and Francesco Ferrara from Cava de Tirreni‐Amalfi Coast University Hospital of Salerno for the Italian population study. The aortic root diameters were measured at end‐diastole at the Ao‐a, Ao‐s, and Ao‐asc using the leading edge to leading edge method (Figure 2) [5, 24], and the values were averaged over three cardiac cycles.

### 5.3 Mathematical Analysis

To make the two different ethnic groups more comparable, the nearest neighbor method of the propensity score was applied at a 1:1 ratio of Chinese and Italians to match the gender and age between the two populations. As a result, a total of 910 Chinese and 910 Italian subjects (408 men and 502 women, aged 45.5 ± 13.9 years with a range of 18–79 years for both populations) were finally selected for data analysis. The Chinese, Italian, and the combined Chinese–Italian populations were each randomly divided at a ratio of 7:3 into group A for the construction of OMAM equations and group B for the validation of OMAM equations.

The OMAM equations were constructed in group A and tested in group B in each of the Chinese, Italian, and combined Chinese–Italian populations. The uncorrected value (Yu) of each aortic diameter was plotted against age, height, and weight in group A, which showed a nonlinear correlation. We assumed that the relationship between the predicted value of each aortic diameter (Yp) and the biometric variables of age, height, and weight follows an exponential equation: Yp = *a* × age*^x^* × height*^y^* × weight*^z^*, where *a* is the allometric scaling constant, and *x*, *y*, and *z* are the exponents of the independent variables of age, height, and weight, respectively. Since there were significant differences in aortic dimensions between men and women, gender was introduced into the above equation as a dummy variable (man = 0, woman = 1), and the above equation evolves into: Yp = *a* × *b* ^gender^ × age*^x^* × height*^y^* × weight*^z^*, where *b* is a constant related to gender. Since both BMI and BSA are calculated from height and weight, they were not added as independent variables into the equations to avoid confounding interactions with the original values of height and weight. In order to facilitate linear regression analysis by statistical software, the above exponential equation was transformed into a linear equation by performing logarithmic transformation on both sides: Ln(Yp) = Ln(*a*) + gender Ln(*b*) + *x* Ln(age) + *y* Ln(height) + *z *Ln(weight). A stepwise multivariable linear regression analysis was performed to determine the variables entering the formula, as well as the values of scaling constants and exponents. The variables that correlated significantly with Yp would remain in the above exponential equation, while the variables that did not correlate significantly with Yp would be automatically excluded from the equation. Then the variables and values were substituted into the above formula to calculate the value of Yp, Yp = *a* × *b* ^gender^ × age*^x^* × height*^y^* × weight*^z^*. The corrected value (Yc) of aortic diameter was defined as the ratio of Yu to Yp as: Yc = Yu/Yp [7, 10]. In addition, the aortic measurements were also indexed with BSA as recommended by current guidelines.

As reported in our previous studies [7, 25], the criterion for successful correction of cardiovascular measurements was as follows: (1) an absence of statistically and biologically significant residual correlations (|*r*| > 0.20, *p *< 0.05) between Yc and age, gender, or body size variables, and (2) a maintenance of significant correlations (*p *< 0.05) between Yc and Yu values. Only when the two criteria are met can the correction be considered successful. Overcorrection was defined as *r *< 0 and *p *< 0.05, indicating that the correlation between the corrected ARD and body size variables shifted from positive to negative or the difference between genders was inverted. Undercorrection was defined as *r *> 0 and *p *< 0.05, indicating that there still existed residual correlations between the corrected ARD and body size variables [26, 27]. To facilitate the clinical application of the OMAM approach, we developed a web‐based calculator. When a user inputs the demographic parameters of a given subject, including age, gender, height, and weight, as well as the measured ARD into the calculator, the predicted and OMAM‐corrected ARD will be automatically provided.

### 5.4 Inter‐ and Intraobserver Variabilities

To verify the reproducibility of aortic measurements, the diameters of Ao‐a, Ao‐s, and Ao‐asc were remeasured in 50 randomly selected subjects from the Chinese population. The interobserver variability was assessed between two investigators (Xiangyun Chen and Andreina Carbone), and the intraobserver variability was assessed by Xiangyun Chen. The Bland–Altman method was employed to analyze the inter‐ and intraobserver variabilities, and ICCs were calculated.

### 5.5 Statistical Analysis

The Kolmogorov—Smirnov test was used to assess the normality of data distribution. For normally distributed parameters, data were expressed as mean ± SD. An independent‐samples unpaired *t*‐test was performed to analyze the differences between genders and between Chinese and Italians. One‐way ANOVA was applied to compare the mean values among three age groups. Bivariate Pearson correlation analyses were used to analyze the correlations between the corrected and uncorrected values, as well as biometric variables. The inter‐ and intraobserver variabilities of aortic measurements were evaluated using Bland–Altman analysis. Statistical analysis was performed using SPSS version 25.0 (SPSS, Inc, Chicago, IL), and a two‐tailed *p *< 0.05 was considered statistically significant.

## Author Contributions

C.Z., E.B., and Y.Z. conceived and designed this study. X.H., X.C., F.F., A.C., and P.S. analyzed and measured echocardiographic data. Y.Z. and X.Z. provided a mathematical analysis model. S.R., M.F., and O.V. conceived the study and offered methodology support. G.Y. and X.H. wrote the original draft. Y.Z. and M.Z. reviewed and edited the manuscript. All authors have read and approved the final manuscript.

## Funding

This work was supported by grants of National Natural Science Foundation of China (82030051, 82430015, 82241203), State Key R&D Program of China (2021YFF0501403, 2022YFC3602403), Taishan Scholars Program of Shandong Province (ts201511091), and Qingdao Science and Technology Project (25‐1‐5‐smjk‐9‐nsh).

## Ethics Statement

The EMINCA study was registered at the Chinese Clinical Trial Registry (http://www.chictr.org; trial registration number: ChiCTR‐OCS‐12002119). It was conducted in 43 collaborating laboratories across the Chinese mainland and approved by the ethical committees of all collaborating hospitals (approval no. 12144). The Italian study was conducted in two hospitals in Italy within the framework of the RIGHT‐Net International Network study, in accordance with the Declaration of Helsinki. It was approved by the ethical committees (protocol No. 110486, and approval no. 84 r.p.s.o‐2‐12‐2015). The Italian study was registered at ClinicalTrials.gov (identifier: NCT03041337). Written informed consent was obtained from all volunteers participating in these two studies.

## Conflicts of Interest

The authors declare no conflicts of interest.

## Consent

The written informed consent was obtained from all volunteers participating in this study.

## Supporting information




**Table S1** Demographic characteristics of the study populations
**TABLE S2** The aortic dimensions stratified by gender and age in the Chinese population
**TABLE S3** The aortic dimensions stratified by gender and age in the Italian population
**Table S4** Comparisons of demographic characteristics between group A and group B in the Chinese, Italian, and combined populations
**Table S5** Correlations of aortic dimensions with physiological variables in group B of the combined Chinese–Italian population

## Data Availability

The data are available from the corresponding authors upon reasonable request.
