# Supplementary material for: Comparison of Body Surface Area and Optimized Multivariate Allometric Model Approaches in Correcting Echocardiographic Aortic Dimensions for Physiological Variances: An International and Multicenter Study (CITED I)
Source: MedComm (2020). 2026 May 29;7(6):e70788. doi: 10.1002/mco2.70788 (PMC13239753; doi:10.1002/mco2.70788)
Supplement: Supplementary file 1 — Table S1 Demographic characteristics of the study populations TABLE S2 The aortic dimensions stratified by gender and age in the Chinese population TABLE S3 The aortic dimensions stratified by gender and age in the Italian population Table S4 Comparisons of demographic characteristics between group A and group B in the Chinese, Italian, and combined populations Table S5 Correlations of aortic dimensions with physiological variables in group B of the combined Chinese–Italian population [file MCO2-7-e70788-s001.docx]

**Comparison of Body Surface Area and Optimized Multivariate Allometric Model Approaches in Correcting** **Echocardiographic Aortic Dimensions for Physiological Variances：an International and Multicenter Study (CITED I)**

***Running head:*** *Correction model of aortic dimensions*

Guihua Yao^1,2^, Xiaoxia Hu^2^, Xiangyun Chen^2^, Xueying Zeng^3^, Francesco Ferrara^4^, Andreina Carbone^5,6^, Salvatore Rega^5^, Monica Franzese^7^, Pin Sun^8^, Mei Zhang^1^, Olga Vriz^9^, Cheng Zhang^1*^, Eduardo Bossone^5,10*^, Yun Zhang^1*^

^1^State Key Laboratory for Innovation and Transformation of Luobing Theory; Key Laboratory of Cardiovascular Remodeling and Function Research, Chinese Ministry of Education, Chinese National Health Commission，Chinese Academy of Medical Sciences and Shandong Province; Department of Cardiology, Qilu Hospital of Shandong University, Jinan, China

^2^Department of Cardiology, Qilu Hospital of Shandong University (Qingdao), Qingdao, China

^3^School of Mathematical Sciences, Ocean University of China, Qingdao, China

^4^Heart Department, University Hospital of Salerno, Salerno, Italy

^5^Department of Public Health, University of Naples "Federico II", Naples, Italy

^6^Unit of Cardiology, University of Campania "Luigi Vanvitelli", Naples, Italy

^7^IRCCS SYNLAB SDN, Naples, Italy

^8^Department of Echocardiography, The Affiliated Hospital of Qingdao University, Qingdao, China

^9^Department of Cardiology and Emergency, San Antonio Hospital, San Daniele Del Friuli, Udine, Italy

^10^Heart Centre of Excellence, King Faisal Specialist Hospital & Research Center, Riyadh, Saudi Arabia.

The first three authors contributed equally to this work.

^*^**Correspondence**: Yun Zhang, Department of Cardiology, Qilu Hospital of Shandong University, Jinan, China, E-mail:zhangyun@sdu.edu.cn; Eduardo Bossone, Department of Public Health, University of Naples "Federico II", Naples, Italy, E-mail: [eduardo.bossone@unina.it](mailto:eduardo.bossone@unina.it), or Cheng Zhang, Department of Cardiology, Qilu Hospital of Shandong University, Jinan, China, E-mail:zhangc@sdu.edu.cn

**Supplementary Information**

**Example of construction of OMAM equation for Ao-a in the Chinese population**

Taking the construction of the OMAM equation for Ao-a in the Chinese population as an example, the relationship between the predicted value of Ao-a (Yp) and the biometric variables of gender, age, height, and weight was assumed to follow an exponential equation: Yp = a·b^gender^·age^x^·height^y^·weight^z^, where a is the allometric scaling constant, b is a constant related to gender, and x, y, and z are the exponents of the variables age, height, and weight, respectively. To facilitate linear regression analysis using statistical software, the above exponential equation was transformed into a linear equation by taking the logarithmic transformation on both sides: Ln(Yp) = Ln(a) + gender·Ln(b) + x·Ln(age) + y·Ln(height) + z·Ln(weight). A stepwise multivariable linear regression analysis was conducted to determine which variables would be included in the formula, and the scaling constants and exponents of these included variables were calculated. Our results showed that gender, age, and weight were significantly correlated with Yp and remained in the above exponential equation, whereas height was not significantly correlated with Yp and was automatically excluded from the above equation. The values of the scaling constants and exponents were also determined as follows: a = 9.593, b = 0.943, x = 0.054, and z = 0.142. Then, these variables and the calculated values were substituted into the above formula to calculate the value of Yp, and the OMAM equation of Yp for Ao-a was constructed as: Yp = 9.593·0.943^(man = 0, woman = 1)^·age^0.054^·weight^0.142^. The corrected value (Yc) of Ao-a was defined as: Yc = uncorrected Ao-a/Yp = uncorrected Ao-a/9.593·0.943^(man = 0, woman = 1)^·age^0.054^·weight^0.142^.

**Table S1** Demographic characteristics of the study populations

|  | **Chinese**  **(n=910)** | **Italian**  **(n=910)** | ***p*-value** |
| --- | --- | --- | --- |
| Age (yrs) | 45.5±13.9 | 45.5±13.9 | 1.000 |
| Men (%) | 44.8% | 44.8% | 1.000 |
| Height (cm) | 165.1±8.1 | 168.6±9.4 | <0.001 |
| Weight (kg) | 61.6±9.6 | 68.9±11.7 | <0.001 |
| BSA (m^2^) | 1.68±0.16 | 1.78±0.20 | <0.001 |
| BMI (kg/m^2^) | 22.5±2.3 | 24.2±2.8 | <0.001 |
| SBP (mmHg) | 118.0±10.4 | 122.5±11.5 | <0.001 |
| DBP (mmHg) | 75.2±7.1 | 75.8±8.3 | 0.136 |

BMI, body mass index; BSA, body surface area; SBP, systolic blood pressure; DBP, diastolic blood pressure.

**TABLE S2** The aortic dimensions stratified by gender and age in the Chinese population

| **Parameters** | **Men (n=408)** | | | | **Women (n=502)** | | | |
| --- | --- | --- | --- | --- | --- | --- | --- | --- |
|  | **18-40 y**  **(n=158)** | **41-65 y**  **(n=220)** | **>65 y**  **(n=30)** | **Total**  **(n=408)** | **18-40 y**  **(n=187)** | **41-65 y**  **(n=275)** | **>65 y**  **(n=40)** | **Total**  **(n=502)** |
| Ao-a (mm) | 21.1±2.2 | 21.5±2.5 | 21.5±2.1 | 21.4±2.4 | 19.2±2.1^**^ | 20.0±2.4^**^ | 20.5±2.6^*^ | 19.7±2.3^**^ |
| Ao-s (mm) | 28.8±2.8 | 30.8±3.2 | 31.4±2.6^*^ | 30.0±3.2 | 26.5±3.2^**^ | 28.1±3.1^**^ | 28.0±3.3^***^ | 27.5±3.2^**^ |
| Ao-asc (mm) | 26.4±3.1 | 28.4±3.7 | 30.0±2.8^*^ | 27.7±3.6 | 24.4±3.5^**^ | 26.6±3.3^**^ | 26.5±3.0^***^ | 25.8±3.5^**^ |
| Ao-a/BSA (mm/m^2^) | 11.6±1.4 | 12.1±1.6 | 12.5±1.4^*^ | 11.9±1.5 | 12.2±1.4^**^ | 12.7±1.6^**^ | 13.4±1.7^***^ | 12.6±1.6^**^ |
| Ao-s/BSA (mm/m^2^) | 15.8±1.6 | 17.3±2.0 | 18.3±1.5^*^ | 16.8±2.0 | 16.9±2.2^**^ | 17.8±2.0^**^ | 18.4±2.7^*^ | 17.5±2.2^**^ |
| Ao-asc/BSA(mm/m^2^) | 14.5±1.7 | 15.9±2.1 | 17.5±1.6^*^ | 15.5±2.1 | 15.5±2.2^**^ | 16.9±2.2^**^ | 17.4±2.2^*^ | 16.4±2.3^**^ |

Ao-a, aortic annular diameter; Ao-s, aortic sinus diameter; Ao-asc, proximal ascending aortic diameter.

^*^*p* < 0.05 inter-age group comparisons according to gender (ANOVA);

^**^*p* < 0.05 compared with men stratified by age;

^***^*p* < 0.05 inter-age group comparisons according to gender and compared with men stratified by age.

**TABLE S3** The aortic dimensions stratified by gender and age in the Italian population

| **Aortic size** | **Men (n=408)** | | | | **Women (n=502)** | | | |
| --- | --- | --- | --- | --- | --- | --- | --- | --- |
|  | **18-40 y**  **(n=158)** | **41-65 y**  **(n=220)** | **>65 y**  **(n=30)** | **Total**  **(n=408)** | **18-40 y**  **(n=187)** | **41-65 y**  **(n=275)** | **>65 y**  **(n=40)** | **Total**  **(n=502)** |
| Ao-a (mm) | 20.6±2.0 | 21.0±2.0 | 22.5±2.0^*^ | 21.0±2.0 | 18.4±1.5^**^ | 18.9±1.5^**^ | 19.6±1.6^***^ | 18.8±1.6^**^ |
| Ao-s (mm) | 30.4±3.2 | 32.8±3.2 | 35.0±3.4^*^ | 32.0±3.5 | 26.7±2.4^**^ | 29.4±2.7^**^ | 31.3±3.3^***^ | 28.5±3.0^**^ |
| Ao-asc (mm) | 27.3±3.1 | 30.9±3.5 | 33.0±3.8^*^ | 29.7±3.9 | 25.2±2.6^**^ | 28.3±2.6^**^ | 30.4±3.3^***^ | 27.3±3.2^**^ |
| Ao-a/BSA (mm/m^2^) | 10.7±1.5 | 11.0±1.2 | 11.9±1.3^*^ | 11.0±1.3 | 11.2±1.2^**^ | 11.3±1.1^**^ | 11.9±1.1^*^ | 11.3±1.1^**^ |
| Ao-s/BSA (mm/m^2^) | 15.8±2.2 | 17.2±2.0 | 18.6±2.5^*^ | 16.8±2.2 | 16.2±1.8 | 17.6±1.9^**^ | 18.9±2.0^*^ | 17.2±2.0^**^ |
| Ao-asc/BSA(mm/m^2^) | 14.2±2.1 | 16.2±2.1 | 17.5±2.3^*^ | 15.5±2.4 | 15.3±1.9^**^ | 17.0±1.8^**^ | 18.4±2.0^*^ | 16.5±2.1^**^ |

Ao-a, aortic annular diameter; Ao-s, aortic sinus diameter; Ao-asc, proximal ascending aortic diameter.

^*^*p* < 0.05 inter-age group comparisons according to gender (ANOVA);

^**^*p* < 0.05 compared with men stratified by age;

^***^*p* < 0.05 inter-age group comparisons according to gender and compared with men stratified by age.

**Table S4** Comparisons of demographic characteristics between group A and group B in the Chinese, Italian, and combined populations

| **Parameters** | **Chinese** | | | **Italian** | | | **Combined** | | |
| --- | --- | --- | --- | --- | --- | --- | --- | --- | --- |
|  | **Group A**  **(n=637)** | **Group B**  **(n=273)** | ***p-value*** | **Group A**  **(n=637)** | **Group B**  **(n=273)** | ***p-value*** | **Group A**  **(n=1274)** | **Group B**  **(n=546)** | ***p-value*** |
| Age (yrs) | 45.4±13.8 | 45.6±14.2 | 0.910 | 45.6±14.2 | 45.2±13.2 | 0.684 | 45.5±14.0 | 45.4±13.7 | 0.835 |
| Height (cm) | 164.9±7.9 | 165.5±8.6 | 0.356 | 168.3±9.4 | 169.0±9.5 | 0.305 | 166.6±8.9 | 167.3±9.2 | 0.176 |
| Weight (kg) | 61.4±9.5 | 62.1±9.8 | 0.322 | 68.7±11.6 | 69.4±11.9 | 0.471 | 65.1±11.2 | 65.7±11.5 | 0.262 |
| BMI (kg/m^2^) | 22.5±2.4 | 22.6±2.3 | 0.609 | 24.1±2.8 | 24.2±2.9 | 0.914 | 23.3±2.7 | 23.4±2.7 | 0.696 |
| BSA (m^2^) | 1.67±0.16 | 1.68±0.17 | 0.294 | 1.78±0.19 | 1.79±0.21 | 0.189 | 1.72±0.18 | 1.74±0.20 | 0.107 |
| SBP (mmHg) | 117.6±10.5 | 118.7±10.1 | 0.152 | 122.6±11.6 | 122.4±11.4 | 0.873 | 120.2±11.4 | 120.6±10.9 | 0.419 |
| DBP (mmHg) | 75.1±7.0 | 75.6±7.3 | 0.398 | 75.7±8.3 | 76.0±8.2 | 0.678d | 75.4±7.7 | 75.8±7.8 | 0.390 |

BMI, body mass index; BSA, body surface area; SBP, systolic blood pressure; DBP, diastolic blood pressure.

**Table S5** Correlations of aortic dimensions with physiological variables in group B of the combined Chinese-Italian population

| **Aortic size** | **Mean values**  **(mean ± SD)** | **Correlations with uncorrected and corrected values (*r, p values*)** | | | | | |
| --- | --- | --- | --- | --- | --- | --- | --- |
|  |  | **Uncorrected value** | **Age** | **Height** | **Weight** | **BSA** | **BMI** |
| Ao-a (mm) | 20.099±2.310 | - | 0.144, 0.001 | 0.284, <0.001 | 0.308, <0.001 | 0.287, <0.001 | 0.190, <0.001 |
| Ao-s (mm) | 29.256±3.659 | - | 0.286, <0.001 | 0.293, <0.001 | 0.412, <0.001 | 0.352, <0.001 | 0.337, <0.001 |
| Ao-asc (mm) | 27.427±3.729 | - | 0.342, <0.001 | 0.187, <0.001 | 0.367, <0.001 | 0.286, <0.001 | 0.376, <0.001 |
| Ao-a/BSA (mm/m^2^) | 11.659±1.581 | 0.610, <0.001 | 0.230, <0.001 | -0.428, <0.001 | -0.476, <0.001 | -0.567, <0.001 | -0.307, <0.001 |
| Ao-s/BSA (mm/m^2^) | 16.952±2.308 | 0.626, <0.001 | 0.369, <0.001 | -0.400, <0.001 | -0.362, <0.001 | -0.492, <0.001 | -0.162, <0.001 |
| Ao-asc/BSA (mm/m^2^) | 15.907±2.384 | 0.688, <0.001 | 0.410, <0.001 | -0.437, <0.001 | -0.338, <0.001 | -0.482, <0.001 | -0.089, 0.040 |
| Ao-a/(2.948·0.928^(man=0, woman=1)^·age^0.060^·Height^0.338^) | 1.004±0.106 | 0.890, <0.001 | 0.064, 0.135 | -0.061, 0.153 | -0.002, 0.971 | -0.027, 0.532 | 0.056, 0.189 |
| Ao-s/(2.689·0.950^(man=0, woman=1)^·age^0.137^·Height^0.210^ ·weight^0.198^) | 0.997±0.105 | 0.808, <0.001 | -0.002, 0.969 | -0.061, 0.154 | -0.047, 0.272 | -0.069, 0.108 | -0.013, 0.762 |
| Ao-asc/(4.375·oi.974^(man=0, woman=1)^ ·age^0.180^·weight^0.280^) | 1.002±0.116 | 0.812, <0.001 | -0.052, 0.232 | -0.042, 0.331 | -0.032, 0.463 | -0.053, 0.220 | -0.006, 0.883 |

Ao-a, aortic annular diameter; Ao-s, aortic sinus diameter; Ao-asc, proximal ascending aortic diameter; BSA, body surface area; BMI, body mass index.
